# Audience emotional appraisals and interaction-weighted salience of short-video communication in international contexts: evidence from Haidilao dance videos on Douyin and TikTok

**DOI:** 10.3389/fpsyg.2026.1931735

**Published:** 2026-10-16

**Authors:** Yizhou Zhang, Li Li

**Affiliations:** 1 International Exchange and Cooperation Office, Sichuan University of Media and Communications, Chengdu, China; 2 Research Center of Film, Television and Theatre of Zhejiang Province, Communication University of Zhejiang, Hangzhou, China

**Keywords:** emotional labor and service boundaries, expressed audience appraisal, Haidilao dance videos, interaction-weighted analytical salience, international communication, short-video platforms

## Abstract

This study examines expressed audience appraisals and recorded engagement in Haidilao dance-video comments from two platform-language environments. The retained corpus contains 9,323 records: 5,199 from Douyin and 4,124 from TikTok after removal of 331 duplicate export records. Frozen multilingual rules assign five-point valence, five-point intensity, and six dominant categories. Agreement with reference labels in a 300-record category-balanced audit is weighted *κ* = 0.794 for valence, weighted *κ* = 0.837 for intensity, and *κ* = 0.848 for category. The internal audit includes 34 records that overlap rule-development examples by identifier or exact text. Performer/service appreciation comprises 23.2% of Douyin and 26.9% of TikTok records and encompasses service, appearance, and performance appraisals. Interaction-weighted patterns are sensitive to popular comments. Douyin Stage-2 reply-weighted negative valence falls from 26.8 to 3.9% after 99th-percentile weight capping. The TikTok source-key reanalysis uses 72 adjacent-window pairs from eight source videos. Two associations have CR1-adjusted t-based 95% intervals excluding 1, but all six delete-one-source jackknife intervals include 1. The findings describe machine-coded expression and cumulative interaction concentration; they do not establish individual psychological change, causal effects, or population-level platform differences.

## 1 Introduction

Short-video platforms transform offline service interaction into internationally circulating public content that can be watched, replicated, and evaluated. Service employees’ bodily performances are repositioned from one component of the dining experience into content resources distributed through international short-video communication (Kaye et al., 2021; Papacharissi, 2015). Haidilao service dances are analytically valuable because audience appraisals become publicly observable in comment text across different platform-language environments. Commenters may identify the dances as joyful, cute, curious, cringe, or enthusiastic service, while also interpreting them as employee emotional labor, customer boundary crossing, or organized performance. The case therefore illustrates how audiences appraise an emotionally ambiguous service event and how these appraisals are expressed and discussed in comment environments (Grandey and Gabriel, 2015; Hochschild, 1983).

Appraisal theory holds that audience emotion depends on interpretations of situational context, normative appropriateness, responsibility, and possible consequences (Lazarus, 1991; Scherer, 2001). In short-video comment sections, such emotional interpretation forms a public social-information environment through comments and interaction indicators. Other users’ emotional expressions can influence subsequent viewers’ judgments and modes of participation, and comment valence can affect audience attitudes, emotions, and behavioral intentions (Chen and Xia, 2024; van Kleef, 2009). These theories motivate examining expressed appraisal alongside recorded social feedback. The present data comprise comment content and cumulative engagement.

Douyin and TikTok have related technological origins but different content ecologies, market arrangements, and user practices (Kaye et al., 2021). In the Haidilao case, Huang and Zainol (2025) compared video-level marketing outcomes and aggregate interaction, but comment-level audience appraisal, language composition, like-based interaction concentration, and reply-based conversation remain less examined. The present study therefore uses platform-language environment as the unit of contextual comparison and focuses on expressed audience appraisal in international short-video communication.

Accordingly, this study addresses three progressive questions. First, how do the six dominant appraisal categories differ between the Chinese-language Douyin and multilingual TikTok retained corpora, and which categories receive greater like- and reply-weighted analytical salience? Second, how do pooled positive valence, negative valence, and high emotional intensity vary across relative comment-sequence stages? Third, within the eight observed TikTok source videos, how are interaction-weighted affective signals in an earlier ordered comment window associated with like and reply rates per retained comment in the next ordered window under a source-video-clustered specification?

The study analyzes comment text, dates, likes, replies, frozen rule-based labels, and available source keys. The eight TikTok source keys define the source-video units; T01–T08 are aliases for those keys in the source crosswalk. D01–D08 denote the eight original Douyin source videos. G01–G08 are secondary balanced groups used to organize and classify Douyin comments in view of unequal comment counts across the source videos. A G group may contain comments from multiple D01–D08 source videos. The formal corpus contains 9,323 retained records. Analyses use frozen machine labels, and the human-reference audit assesses coding agreement.

Research on audiences’ expressed appraisals of Haidilao dance videos on short-video platforms mainly involves emotion appraisal, service emotional labor, online comment feedback, platformized communication, and international circulation. Existing work points to three issues: how audiences assign emotional meaning to the same service performance in different platform-language environments, how like and reply weighting changes the analytical prominence of those interpretations, and whether affective themes in earlier comment windows are associated with engagement outcomes in the next ordered windows.

This study treats Chinese-language Douyin and multilingual TikTok as two platform-language comment environments. Their differences may reflect language, platform ecology, user practice, video composition, and viewing experience; they cannot be equated with commenters’ nationality, native language, or stable cultural identity (Kaye et al., 2021). Emotion appraisal refers here to interpretations expressed in comment text about employee movement, service roles, customer participation, and interactional atmosphere. Pleasure, curiosity, embarrassment, sympathy, and labor concerns are coded as expressed appraisals.

Employee behavior in Haidilao dance videos can also be understood through emotional labor. Emotional labor is the process by which employees regulate and display emotions in order to meet work-role requirements, and it can also be understood as a mechanism of emotion regulation in the workplace (Hochschild, 1983; Grandey, 2000). Once such emotional and bodily display is recorded, circulated, and commented on through short-video platforms, it enters the domain of platformized service performance. Research also associates emotional labor with employee outcomes (Humphrey, 2023), which motivates distinguishing labor-related appraisals from entertainment reactions.

As shown in Table 1, the coding framework contains three dimensions. Emotional valence is scored from 1 (extreme negative) to 5 (extreme or amplified positive), emotional intensity is scored from 1 (no clear affective signal) to 5 (extreme amplified expression), and dominant category is coded as entertainment/humor, cringe/social discomfort, curiosity/spectacle, performer/service appreciation, emotional labor/service boundaries, or other/participatory context. Performer/service appreciation includes service/staff-performance appraisal, performer appearance admiration, and general positive performance appreciation.

**Table 1:** Coding dimensions and operational categories.

Coding dimension	Operational definition
Emotional valence	1 = extreme negative; 2 = negative; 3 = neutral/mixed/insufficient direction; 4 = positive; 5 = extreme or amplified positive expression.
Emotional intensity	1 = no clear affective signal; 2 = low/weak expression; 3 = moderate evaluation or expressive emoji; 4 = high/amplified expression; 5 = extreme amplified expression or sustained unambiguous repetition.
Dominant category 1	Entertainment/Humor
Dominant category 2	Cringe/Social Discomfort
Dominant category 3	Curiosity/Spectacle
Dominant category 4	Broad performer/service appreciation: 4a service/staff performance, 4b performer appearance, 4c general performance appreciation, or mixed 4a + 4b. Appearance alone is not service-quality evidence.
Dominant category 5	Emotional Labor/Service Boundaries
Dominant category 6	Other/Participatory Context

Emotion recognition research shows that individuals can usually understand others’ emotional cues to some extent, but the specific recognition results and attached meanings are shaped by contextual experience, social norms, and language concepts (Elfenbein and Ambady, 2002; Mesquita and Frijda, 1992). For Haidilao dance videos, this perspective motivates examining how commenters describe service intentions, role relations, customer participation, and performance appropriateness.

Cross-cultural emotion-perception research suggests that perceivers can attribute additional, unintended emotions to the same cue (Fang et al., 2019). This motivates examining the range of interpretations expressed about service dances.

Service research further shows that positive service-performance appraisal does not necessarily receive uniform interpretation. High service attentiveness may be understood in one comment context as care, warmth, and high-quality service, whereas in another it may raise questions about service motive, service boundaries, or employee condition (Liu et al., 2019). Customers’ recognition of employees’ emotional display strategies also affects judgments of service authenticity, service quality, and interactional relationships (Groth et al., 2009).

Existing research suggests that the same emotional cue and service behavior may receive different interpretations across platform-language environments. Relatively little work has examined how platformized service performance is interpreted through expressed audience appraisals in international short-video communication while separating platform context from national or cultural identity.

*RQ1*: Do emotion-interpretation themes differ between the Chinese-language Douyin and multilingual TikTok samples? Which themes receive greater like- and reply-weighted analytical salience?

Audience response does not occur only at the level of individual viewing; it is also situated within comment environments and platform mechanisms. The emotions-as-social-information perspective argues that others’ emotional expressions transmit cues about events, relationships, and norms to subsequent observers (van Kleef, 2009). Online comment research also shows systematic associations between comment valence and audiences’ attitudes, emotions, and behavioral intentions toward original content (Chen and Xia, 2024).

Prior experimental research indicates that video comments can alter viewers’ emotional responses to the same content. On short-video platforms, highly liked, highly replied-to, or prominently ranked comments may be more likely to enter users’ field of view. Emotion interpretation in comment sections therefore should not be measured only by frequency of occurrence (Krämer et al., 2021). In the present study, like and reply weights index interaction-concentrated prominence.

Likes and replies may reinforce different types of comment expression. When users receive likes and comments, they may experience different emotional responses and adjust subsequent expressive behavior (Stsiampkouskaya et al., 2021). Like weighting and reply weighting therefore need to be distinguished in the analysis.

Douyin and TikTok share related technological systems but differ in content ecology, user practice, and platformization path (Kaye et al., 2021). Existing cross-platform research on Haidilao mainly compares the content characteristics and public interaction indicators of official-account videos, and has not sufficiently examined comment emotion, reply chains, or temporal evolution (Huang and Zainol, 2025).

Online emotional expression may also vary during the interactional process. Emotional expression in online discussion is dynamically associated with participation (Garcia et al., 2016), and social feedback may be related to later changes in emotional language (Luo et al., 2025). Differences between the two platform comment contexts may therefore appear not only in which themes are present, but also in their relative-order peaks, turns, and declines.

*RQ2*: How do like-weighted positive valence, reply-weighted negative valence, and reply-weighted high emotional intensity vary across five pooled relative comment-sequence stages in the Douyin and TikTok retained corpora?

Early emotional expression in online interaction may provide later users with references for understanding and responding to content. Exposure to emotion on social media is associated with subsequent emotional expression (Ferrara and Yang, 2015). Emotional expression in online interaction may also show accumulation, continuation, or redirection over time (Garcia et al., 2016).

Social feedback can further participate in this process. Research on online video live-chat interaction shows temporal associations between user feedback and subsequent emotional language (Luo et al., 2025). This finding provides a reference for examining prior affective salience and later interactional change in short-video comments.

Interaction-weighted emotion-interpretation themes in an earlier comment window may covary with engagement rates per retained comment in the next ordered window. Those rates may also reflect platform recommendation, account influence, video posting time, popular-comment ranking, and external circulation.

Engagement counts also need to distinguish broad interaction from conversational attention. Like and reply volumes can reflect different forms of participation, while reply volume itself cannot directly represent controversy intensity. Controversial discussion requires separate identification of rebuttal, stance conflict, negative evaluation, or disagreement.

Such ordered-window relationships may depend on the platform-language environment (Kaye et al., 2021). The present source-aware model is restricted to TikTok.

*RQ3*: Within the eight observed TikTok source videos, how are interaction-weighted positive valence, negative valence, and high emotional intensity in an earlier ordered comment window associated with like and reply rates per retained comment in the next ordered window?

Overall, emotion-recognition research has moved from basic-cue recognition toward contextualized understanding of emotional meaning, service research shows that employees’ emotional displays enter customer evaluation, and online-comment research shows that social feedback and interaction indicators can differentiate the prominence of interpretations. The present study therefore locates its contribution in international short-video communication and uses platform-language samples for contextual comparison.

Nevertheless, existing research can still be advanced in several ways. First, audience emotion research has rarely addressed collective interpretation of service performance in platform comment sections. Second, service research has paid limited attention to the psychological evaluation of video-mediated employee performance by non-present publics. Third, interaction research has rarely distinguished how different emotion-interpretation themes are emphasized under like and reply weighting. Fourth, cross-platform research has rarely examined ordered-window associations between affective themes and engagement rates per retained comment while accounting for source-video clustering.

## 2 Materials and methods

### 2.1 Data sources and sample composition

The analysis uses 5,199 retained Douyin records and 4,124 retained TikTok comments. The TikTok duplicate-removal log and source-key multiplicities document 4,455 export records, 331 excess records, and 283 repeated-identifier groups. The recorded procedure retained one record per original comment identifier; the removal log corresponds internally to the 4,124 unique pseudonymous comment keys. Identical text alone was not a deletion criterion. The public release excludes original identifier-bearing exports, limiting independent checks of duplicate equality. Douyin contains 2,005 resolved comment keys, 1,837 records marked ambiguous, and 1,357 marked unresolved. All 5,199 Douyin records are retained; original-ID uniqueness and complete video membership remain unverified. Table 2 summarizes the retained samples and analysis scope.

**Table 2:** Retained corpus, category composition, reference agreement, and analytical scope.

Analysis component	Douyin	TikTok	Validation/note
Retained machine-coded corpus	5,199	4,124	Total *N* = 9,323
Documented TikTok duplicate removals	Not applicable	331 from 4,455	4,124 unique pseudonymous comment keys retained
Observed/missing replies	4,835/364	4,124/0	Missing Douyin replies excluded from primary reply-weighted denominators
Theme 1 Entertainment/Humor	672 (12.9%)	725 (17.6%)	
Theme 2 Cringe/Social Discomfort	55 (1.1%)	4 (0.1%)	
Theme 3 Curiosity/Spectacle	249 (4.8%)	46 (1.1%)	
Theme 4 Performer/Service Appreciation	1,204 (23.2%)	1,108 (26.9%)	
Theme 5 Emotional Labor/Service Boundaries	239 (4.6%)	91 (2.2%)	
Theme 6 Other/Participatory Context	2,780 (53.5%)	2,150 (52.1%)	
Reference agreement audit	207	93	300 records; 50 per machine category pooled
Valence agreement	κw = 0.786	κw = 0.780	Pooled κw = 0.794; exact 89.0%
Intensity agreement	κw = 0.860	κw = 0.783	Pooled κw = 0.837; exact 88.7%
Dominant-category agreement	*κ* = 0.819	*κ* = 0.907	Pooled *κ* = 0.848; exact 87.3%
Observed comment dates	21 Nov 2023–23 Jul 2026	20 Nov 2023–3 Jul 2026	Dates refer to comments, not collection windows
Source-clustered model	Not estimated	8 sources; 72 pairs	Douyin source membership incomplete; TikTok source-key mapping verified
Known development overlap	23 records	11 records	34 audit records by ID or exact text
Audit excluding known overlap	184	82	*N* = 266; *κ* = 0.797/0.825/0.842
Text-evaluable records	5,127	4,008	72/116 non-evaluable records retained

Therefore, Douyin results are interpreted descriptively at the corpus level and are not treated as source-clustered estimates.

During the processing of Douyin comments, substantial differences in comment counts across the eight source videos motivated the construction of secondary balanced groups G01–G08 to reduce the influence of unequal comment volumes on comment organization and classification. G01–G08 do not represent independent source videos; comments within the same G group may originate from multiple D01–D08 source videos. D01–D08 denote the original source videos, although complete comment-level membership is unavailable; G01–G08 support balanced comment-level organization and classification. TikTok source-aware analyses join Source_Comment_Key to the source-key file with a verified one-to-one match for all 4,124 records. The crosswalk assigns T01–T08 by the lexicographic order of the eight source keys and reports their sample sizes: 180, 353, 1,814, 1,443, 26, 143, 107, and 58. Group sizes and source cross-tabulations are provided in the Supplementary materials.

Recorded comment dates span 21 November 2023–23 July 2026 for Douyin and 20 November 2023–3 July 2026 for TikTok. Exact collection dates, search/ranking settings, original video eligibility decisions, and a complete comment inclusion/exclusion log are undocumented. The analyzable sample comprises all retained rows in the two comment tables, including records without interpretable text; no additional engagement threshold or language filter is applied. The corpus is nonprobability-based, and its sampling frame and exposure history cannot be fully reconstructed.

### 2.2 Language composition and machine coding agreement audit

The retained 4,124-record TikTok corpus is multilingual. The de-identified text contains Latin-script, Han-script, Thai-script, Japanese kana/Han-mixed, Hangul, Cyrillic, Arabic/Hebrew, mixed-script, and emoji/symbol-only material. The deterministic coding resources therefore include multilingual lexical and contextual cues for Chinese, English/Latin-general, Vietnamese, Spanish, Indonesian, Korean, Japanese, Thai, and emoji. Language and script cues support text processing. The 230-entry multilingual cue inventory is supplied in the Supplementary files.

The audit workbook contains 300 records: 207 Douyin and 93 TikTok, with 50 records per machine-assigned dominant category across the two platforms combined. The coding workflow proceeded sequentially: retained comments were first processed using the deterministic multilingual rule procedure to generate machine-coded labels. These labels were then reviewed against independently produced human-reference labels by two multilingual communication PhDs, with translation software and dictionaries used when necessary. Ambiguous cases received targeted contextual review, and limited adjustments informed rule refinement before the final labels were frozen for statistical analysis. Comparison with the human-reference labels gives quadratic-weighted Cohen’s κ = 0.794 for valence and 0.837 for intensity, unweighted κ = 0.848 for category, exact agreement of 89.0%, 88.7%, and 87.3%, and category macro-F1 = 0.873. These statistics represent internal agreement after limited rule refinement. In addition, 33 audit identifiers occur in the 400-record Development_Set; identifier or exact-text matching identifies 34 overlapping audit records. Excluding these 34 records leaves 266 and yields *κ* = 0.797, 0.825, and 0.842, respectively.

Category coverage is not balanced separately by platform. TikTok contains only four machine-coded category-2 records and 15 category-5 records in the audit (five and 16 under the reference labels). The small counts limit assessment of rare-category performance. Per-category precision, recall, F1, and confusion matrices are supplied. Because sampling is balanced by machine category, pooled agreement represents internal agreement rather than accuracy under natural corpus prevalence.

### 2.3 Coding of audience emotion and interaction weighting

The coding unit is one retained record. Final labels were generated using HDL-MACHINE-RULES-20260927-v6.0, a deterministic multilingual text-rule procedure. During execution, comment text was first normalized and cleaned, after which the lexicon and rule documentation was used to identify multilingual evaluative, service/performance, labor, discomfort, curiosity, entertainment, and emoji cues while handling local negation. The dominant category was assigned according to a predefined priority structure, and five-point valence and intensity scores were derived from positive and negative appraisal, amplification, repetition, punctuation, and emoji expression. The frozen rules and labels were then used for subsequent statistical analysis and reproducibility.

The lexicon is a cue inventory containing 230 entries (229 unique patterns). Valence anchors range from extreme negative (1), negative (2), neutral/mixed/insufficient directional evidence (3), positive (4), to extreme or amplified positive expression (5). Intensity ranges from no clear affective signal (1), weak expression (2), clear evaluation or expressive emoji (3), strong or amplified expression (4), to extreme or sustained amplified expression (5). The documented theme priority is labor, discomfort, service appraisal, inquiry/spectacle, amusement, then residual context, followed by contextual appreciation handling. Rule evidence and specificity flags are retained per record. Specificity is not a calibrated confidence probability. The five-point outputs are ordinal operational codes, not direct psychometric measurements.

Category 4 comprises 4a service/staff-performance appraisal, 4b performer appearance admiration, 4c general positive performance appreciation, or a mixed 4a + 4b subtype. The separate Strict_Service_Category field retains only 90 Douyin records (1.7%) and 11 TikTok records (0.3%) in category 4, compared with 1,204 (23.2%) and 1,108 (26.9%) under the broad definition. Examples and subtype fields document these distinctions. Records flagged as not text-evaluable (72 Douyin and 116 TikTok) remain in the primary record-based denominators with their residual labels; neutral/insufficient-evidence codes for photos or blank placeholders indicate insufficient text evidence. Text-evaluable-only summaries are supplied as a sensitivity.

Raw proportions use all retained records. Like-weighted salience is the sum of likes + 1 for records in a category divided by that sum for all records in the relevant platform, stage, or window. Reply-weighted salience uses observed replies + 1 over observed reply records only. The 364 missing Douyin reply counts remain missing; primary reply-weighted summaries use 4,835 records, with missing-as-zero assessed separately. Positive valence means scores 4–5, negative valence 1–2, and high intensity 4–5. The optional Class_Balance_Weight fields in the workbooks are not used in the reported figures or models. Interaction weights describe cumulative recorded concentration, not actual exposure, endorsement, or controversy.

### 2.4 Statistical models and analytical strategy

First, category proportions and ordinary interaction-weighted shares were calculated for each retained corpus. Second, records were sorted by comment date and then anonymized comment ID within platform. The zero-based sorted rank r was assigned to stage 1 + floor(5r/N), giving stage sizes 1,040, 1,040, 1,040, 1,040, and 1,039 for Douyin, and 825, 825, 825, 825, and 824 for TikTok. The ID tie-break makes date-level ordering reproducible but does not establish order within a day. A TikTok timestamp-order sensitivity is also supplied. Third, for the TikTok source-key reanalysis, records were sorted within source video by exact recorded timestamp and then anonymized ID, and split into 10 consecutive approximately equal-count windows. If a video’s sample size is not divisible by 10, the earliest windows receive one extra record. This yields 80 windows and 72 adjacent-window pairs. Original timestamp timezone information is not specified in the source-key file.

Six separate negative-binomial NB2 models with log links were fitted: one model for each of three prior-window signals and each of two next-window outcomes (likes or replies). The three signals are like-weighted positive valence, reply-weighted negative valence, and reply-weighted high intensity. The outcome is the sum of observed interaction counts in the next window. Its log mean equals the sum of log (next-window comment count), an intercept, a term for the prior affective share divided by 0.10, a term for log(1 + the same interaction count in the prior window), and a term for prior-window index divided by 10. There are four estimated coefficients per model; no video fixed effects, interactions, or joint inclusion of the three affective signals are used. The next-window comment count is an offset with coefficient fixed at one, so rates are per retained comment, not per viewer exposure or unit of elapsed time. NB2 dispersion is fixed at *α* = 1, giving variance *μ* + *μ*^2^; *α* = 0.5 and 2 are examined as sensitivities. Numerical optimization was checked against the likelihood score and observed Hessian before any estimate was reported.

Uncertainty uses the observed-information sandwich covariance with scores summed within the eight source videos. The CR1 finite-sample multiplier is (8/7) × (71/68), and 95% intervals use the t distribution with seven degrees of freedom. A delete-one-source jackknife sensitivity refits all eight seven-video subsets; its covariance is 7/8 times the sum of outer products of deletion estimates centered at their mean, with *t*(7) intervals around the full-sample coefficient. The jackknife assesses source influence. Eight clusters remain insufficient for dependable large-sample inference, and these corrections do not establish reliable population coverage (Cameron and Miller, 2015). Models are exploratory, with no multiplicity adjustment.

Sensitivity analyses retain the full corpus as the primary analysis. Within each platform and separately for likes and observed replies, weights were capped at their 99th percentile or the 10 largest weights were omitted from that metric’s sensitivity denominator (ties ordered by anonymized ID). Stage boundaries were held fixed for these weight perturbations. The highest-like records were also screened against text and rule evidence, with ambiguous cases reviewed; unresolved cases remained in the final frozen labels. A duplicate replay expands retained TikTok records using their recorded excess-duplicate multiplicities and reruns distributions, stages, and models using the same frozen labels. All models and sensitivity outputs are supplied in the reproducibility package. Cumulative engagement observed at collection cannot establish whether earlier comments received those interactions before later comments were posted.

Generative AI disclosure. Generative AI (ChatGPT5.5 and 5.6 and 6, OpenAI) assisted with rule-development examples and documentation, data-structure checks, analysis-code preparation and checking, numerical verification, figure preparation, translation, language editing, and revision materials. The authors are responsible for checking the underlying data, human-reference provenance, code, figures, citations, and interpretations and for approving the submitted version.

## 3 Results

### 3.1 RQ1 expressed appraisal categories and interaction weighting

As shown in Figure 1, the two platform-language environments differ in raw dominant-category composition and in the analytical salience produced by like and reply weighting.

**Figure 1:**
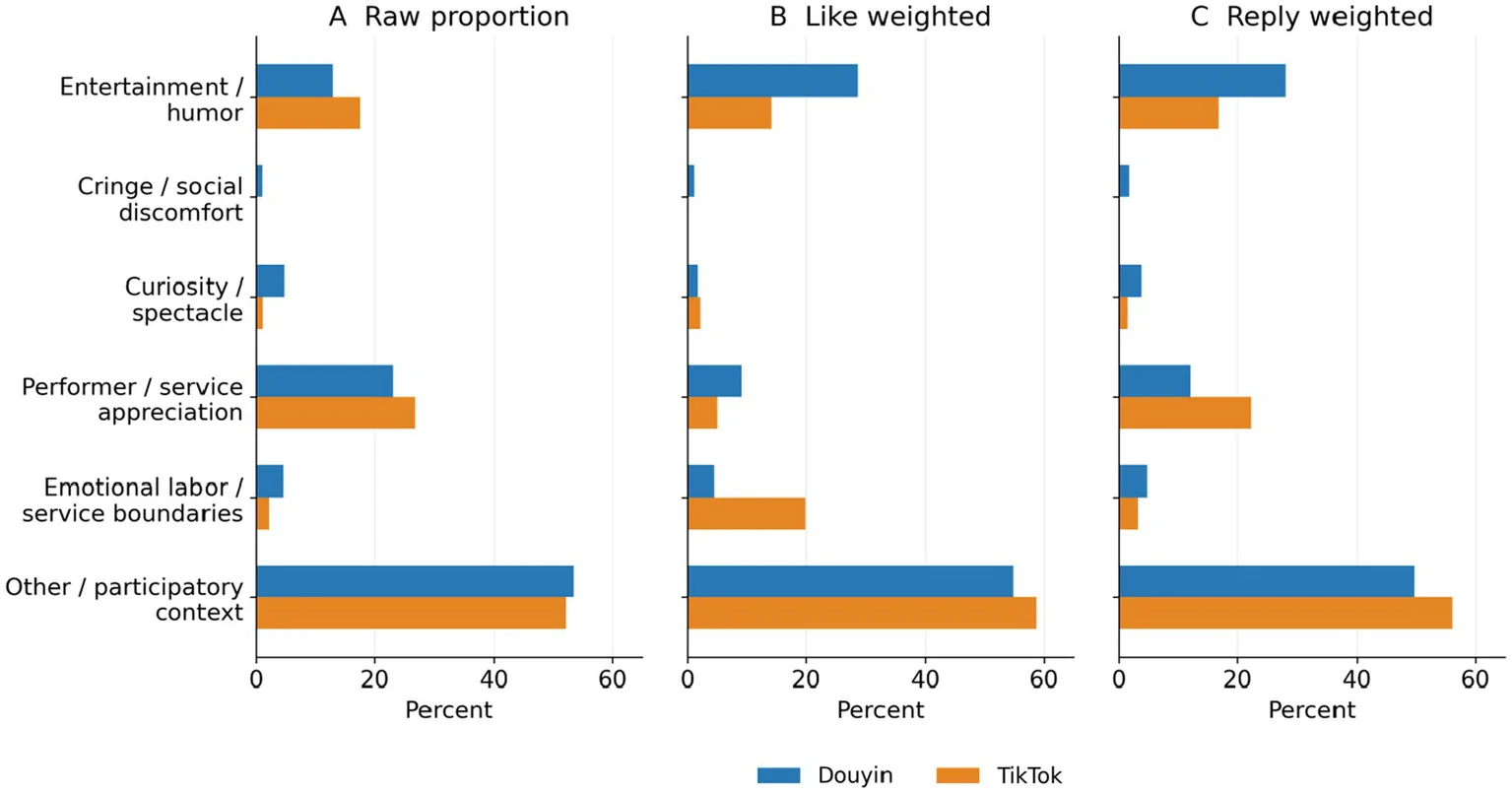
Category proportions and interaction-weighted salience. Panels **(A–C)** show raw, like-weighted, and reply-weighted shares for 5,199 Douyin and 4,124 TikTok records. Weights are likes + 1 and observed replies + 1; primary reply denominators use 4,835 Douyin and 4,124 TikTok records. These are ordinary weights, without G-group or equal-category standardization. Category 4 is broad performer/service appreciation, not service quality alone.

Raw dominant-category distributions differ across the two retained corpora. In Douyin, other/participatory context accounts for 53.5%, performer/service appreciation for 23.2%, entertainment/humor for 12.9%, curiosity/spectacle for 4.8%, emotional labor/service boundaries for 4.6%, and cringe/social discomfort for 1.1%. In TikTok, other/participatory context accounts for 52.1%, performer/service appreciation for 26.9%, entertainment/humor for 17.6%, emotional labor/service boundaries for 2.2%, curiosity/spectacle for 1.1%, and cringe/social discomfort for 0.1%.

Like weighting changes the analytical salience of categories. In Douyin, other/participatory context accounts for 54.8% of like-weighted salience, entertainment/humor for 28.7%, performer/service appreciation for 9.1%, emotional labor/service boundaries for 4.5%, curiosity/spectacle for 1.7%, and cringe/social discomfort for 1.2%. In TikTok, other/participatory context accounts for 58.7%, emotional labor/service boundaries for 19.9%, entertainment/humor for 14.2%, performer/service appreciation for 5.0%, curiosity/spectacle for 2.2%, and cringe/social discomfort for less than 0.1%.

Reply weighting produces a different ordering. In Douyin, other/participatory context accounts for 49.6% of reply-weighted salience, entertainment/humor for 28.2%, performer/service appreciation for 12.0%, emotional labor/service boundaries for 4.7%, curiosity/spectacle for 3.8%, and cringe/social discomfort for 1.7%. In TikTok, other/participatory context accounts for 56.1%, performer/service appreciation for 22.3%, entertainment/humor for 16.8%, emotional labor/service boundaries for 3.2%, curiosity/spectacle for 1.5%, and cringe/social discomfort for 0.1%.

### 3.2 RQ2 pooled comment sequence variation in affective salience

As shown in Figure 2, the three affective indicators follow different trajectories across five pooled relative comment-sequence stages in the retained Douyin and TikTok corpora. The stages contain approximately equal comment counts rather than equal calendar durations.

**Figure 2:**
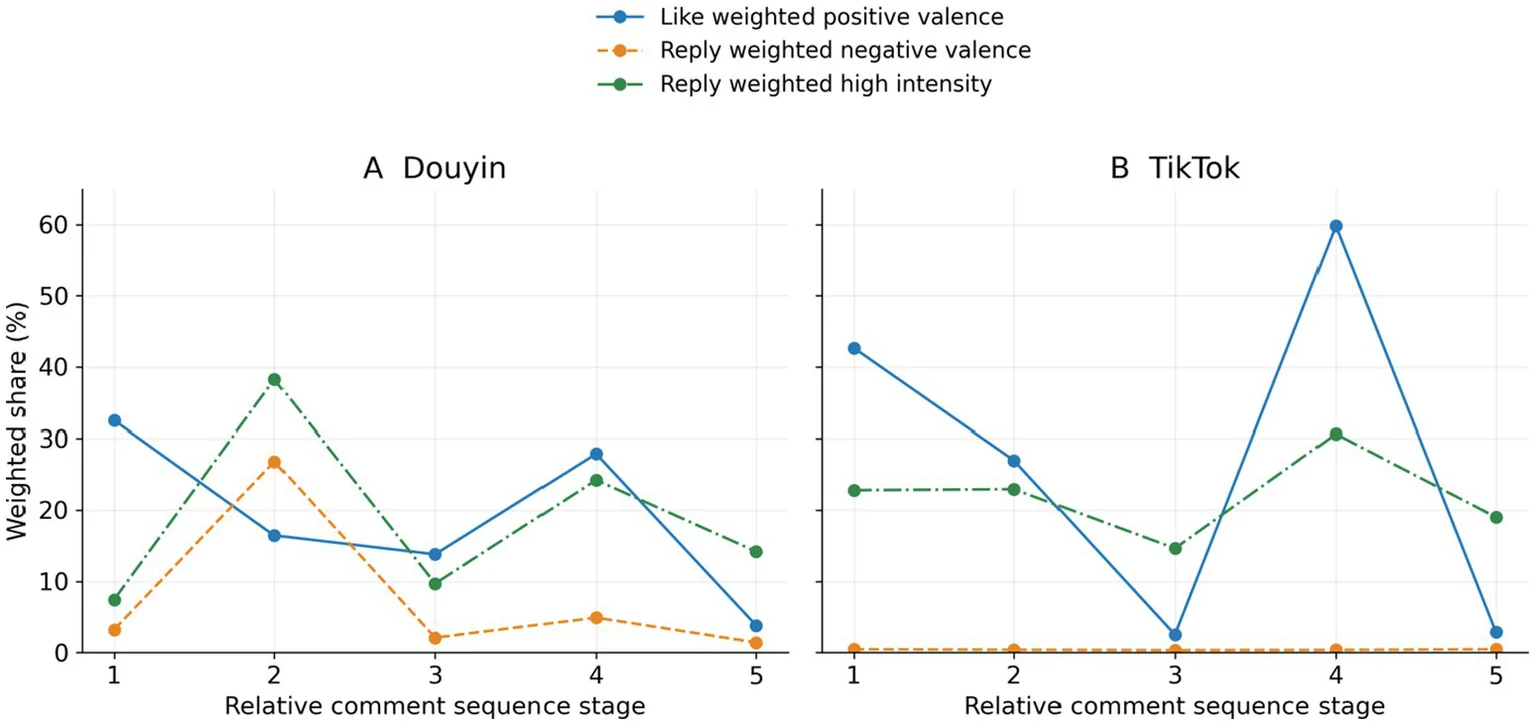
Pooled affective shares across five comment-sequence stages. **(A)** Douyin; **(B)** TikTok. Records are sorted by date and anonymized ID within platform and divided by rank into approximately equal-count stages. Positive valence is scores 4-5, negative valence 1-2, and high intensity 4-5. Curves use ordinary cumulative interaction weights; date ties do not establish within-day chronology. Stages are neither equal-duration nor source-balanced. The Douyin Stage-2 peak attenuates substantially when extreme reply weights are reduced.

In Douyin, like-weighted positive valence is 32.5% in Stage 1, 16.4% in Stage 2, 13.8% in Stage 3, 27.9% in Stage 4, and 3.8% in Stage 5. Reply-weighted negative valence is 3.2%, 26.8%, 2.1%, 4.9%, and 1.4%. Reply-weighted high intensity is 7.4%, 38.3%, 9.7%, 24.2%, and 14.2%.

In TikTok, like-weighted positive valence is 42.7%, 27.0%, 2.5%, 59.8%, and 2.9% across the five stages. Reply-weighted negative valence is 0.44%, 0.37%, 0.31%, 0.34%, and 0.45%, respectively. Reply-weighted high intensity is 22.8%, 22.9%, 14.6%, 30.7%, and 19.0%.

These trajectories describe the pooled retained records. Douyin’s Stage-2 reply-weighted negative share falls from 26.8 to 3.9% under 99th-percentile capping and to 2.6% when the 10 largest reply weights are omitted; high intensity falls from 38.3 to 21.2 and 17.9%, respectively. Thus the prominent Stage-2 peak is strongly concentrated in highly replied-to records. On TikTok, replacing date/ID ordering with exact timestamp ordering changes Stage-4 like-weighted positive valence from 59.8 to 66.4%, showing sensitivity to within-day ordering.

### 3.3 RQ3 ordered window associations on Douyin and TikTok

Figure 3 presents the TikTok source-key ordered-window estimates. Points and intervals represent the six TikTok source-key estimates. The following numerical results report the TikTok source-key reanalysis using all 4,124 retained comments.

**Figure 3:**
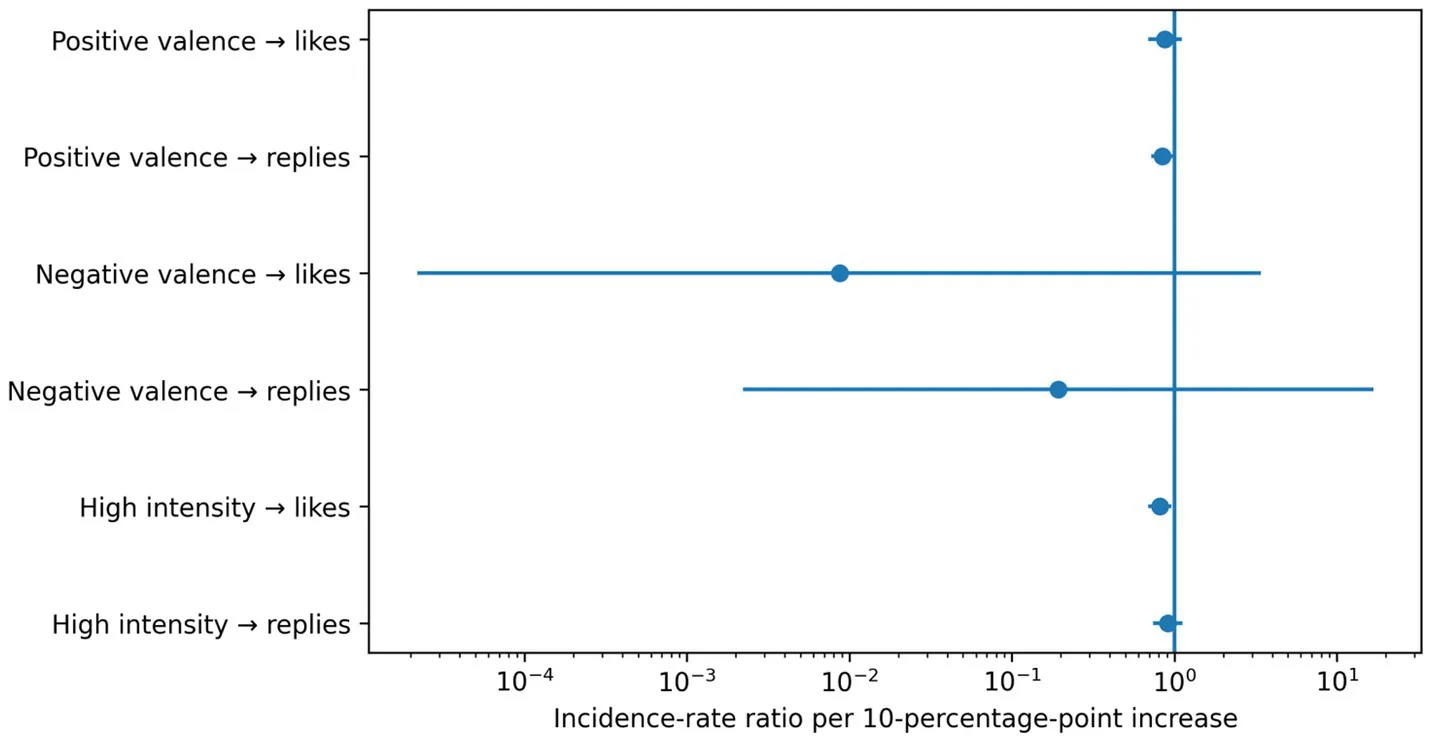
TikTok source-key ordered-window estimates. Points show incidence-rate ratios per 10-percentage-point increase in earlier-window positive valence, negative valence, or high emotional intensity for next-window like or reply counts. Horizontal lines show the corresponding 95% confidence intervals. The analysis includes eight TikTok source videos and 72 adjacent-window pairs. The horizontal axis uses a logarithmic scale, and the dashed vertical line marks an incidence-rate ratio of 1.

Converged TikTok NB2 estimates with CR1 covariance and *t*(7) 95% intervals are: earlier like-weighted positive valence, IRR = 0.872 [0.687, 1.107] for next-window likes and 0.838 [0.717, 0.980] for replies; earlier reply-weighted negative valence, IRR = 0.00868 [0.000022, 3.407] for likes and 0.193 [0.00221, 16.793] for replies. All IRRs are per 10-percentage-point increase in the prior affective share.

Earlier reply-weighted high intensity gives IRR = 0.812 [0.687, 0.959] for next-window likes and 0.911 [0.738, 1.124] for replies. Two CR1/*t*(7) intervals exclude 1.00. However, all six delete-one-source jackknife intervals include 1.00. For the two corresponding associations, jackknife intervals are [0.700, 1.004] and [0.556, 1.185]. Negative-valence estimates are particularly unstable and can reverse direction when one source is omitted. With dispersion fixed at *α* = 0.5, the positive-valence/reply interval also includes 1.00. These results are exploratory and do not support a claim of robust predictive or causal relationships.

The TikTok offset adjusts for the number of retained comments in the following window; model associations are therefore conditional on the retained sample and cumulative counts. For Douyin, G01–G08 were used as secondary balanced processing groups rather than original source-video identifiers; therefore, they were not treated as source-video units in the corresponding reanalysis.

### 3.4 Duplicate replay and interaction weight sensitivity

Replaying the 331 documented duplicates leaves the overall category ordering similar but changes magnitudes. The largest absolute pre/post-deduplication shifts are 1.48 percentage points in raw category shares, 0.24 points in like-weighted shares, and 1.28 points in reply-weighted shares. TikTok Stage-4 like-weighted positive valence changes from 48.1% in the reconstructed duplicate-containing export to 59.8% after deduplication. The high-intensity/next-window-like IRR changes from 0.540 to 0.812 under the same converged *α* = 1 specification.

The 10 largest like weights contribute 48.7% of total like weight in Douyin and 43.2% in TikTok; the 10 largest observed reply weights contribute 39.3 and 5.5%. TikTok emotional labor/service boundaries account for 19.9% of ordinary like-weighted salience, 9.1% after 99th-percentile capping, and 9.0% after omitting the 10 largest like weights. Douyin entertainment/humor changes from 28.7 to 16.2 and 14.5% under the corresponding like-weight sensitivities. Other/participatory context remains the largest category, but several magnitudes and relative rankings change. Missing-as-zero changes any Douyin reply-weighted category share by at most 0.84 percentage points. A targeted human review of the 20 most-liked records slightly refined the machine-coded labels for ambiguous irony, referents, multilingual content, and photo-only cases.

## 4 Discussion

Haidilao dance comments show differences in machine-coded appraisal and interaction concentration across two platform-language environments. Descriptive analyses cover 9,323 retained records. Figure 3 presents the TikTok source-key ordered-window estimates, and the Douyin source-clustered model is not estimated because complete comment-level source membership cannot be verified.

### 4.1 Appraisal categories and construct boundaries

RQ1 shows that both corpora are dominated by other/participatory context and performer/service appreciation, while TikTok contains a higher share of entertainment/humor and Douyin contains higher shares of curiosity/spectacle and emotional labor/service-boundary comments.

The broad performer/service appreciation theme is supported by 1,204 Douyin comments and 1,108 TikTok comments, corresponding to raw prevalences of 23.2 and 26.9%, respectively. This category combines service/staff-performance appreciation, performer appearance admiration, and general positive performance appreciation, extending beyond service-quality evaluation. Under the narrow service-related field, only 90 Douyin and 11 TikTok records remain in category 4. The broad-category result therefore primarily concerns performer or performance appreciation.

Interaction weighting distinguishes textual prevalence from interaction-concentrated analytical salience. In Douyin, other/participatory context remains the largest like- and reply-weighted category, with entertainment/humor and performer/service appreciation also receiving substantial interaction weight. In TikTok, other/participatory context remains dominant under both forms of weighting, while emotional labor/service boundaries receive disproportionate like-weighted analytical salience relative to their raw frequency. This TikTok enrichment attenuates substantially under weight capping and top-10 omission and reflects concentrated attention to a small set of comments.

### 4.2 Comment sequence patterns and highly weighted records

RQ2 shows stage variation under the prespecified date/ID ordering, including an ordinary-weight Stage-2 negative-valence peak in Douyin. That peak attenuates sharply when extreme reply weights are reduced. TikTok has low machine-coded negative-valence shares, but the classifier’s limited rare-category coverage and multilingual ambiguities preclude interpreting this as the absence of discomfort among viewers.

Interaction weighting changes which records dominate a descriptive summary. The observed stage patterns reflect comment content, source composition, arbitrary ordering of date ties, and cumulative engagement concentration. They do not identify temporal changes in individual psychology or the timing of social influence.

### 4.3 Exploratory TikTok ordered window associations

Two associations exclude 1.00 under the CR1/*t*(7) specification: positive valence with next-window reply rate (IRR = 0.838 [0.717, 0.980]) and high intensity with next-window like rate (IRR = 0.812 [0.687, 0.959]). Both intervals include 1.00 under the delete-one-source jackknife. Consequently, the direction of these fitted associations is descriptive, and the evidence does not support a robust inferential claim. The highly uncertain negative-valence coefficients, unequal source sizes, dispersion sensitivity, and cumulative outcome timing further constrain interpretation.

Reply counts indicate recorded conversational engagement, without identifying disagreement or controversy. The NB2 specification allows overdispersion (Hilbe, 2011), but a fixed dispersion assumption and a clustered covariance do not remove omitted source characteristics, sampling bias, or the difficulty of inference from eight videos.

### 4.4 Theoretical and practical implications

The theoretical contribution lies in connecting audience emotional appraisal, emotional-labor perception, and interaction-weighted social-feedback indicators within international short-video communication. First, audience emotion recognition can be studied through the labels commenting publics assign to service performance. Second, platform circulation extends employee performance to non-present publics who evaluate authenticity, labor cost, and organizational responsibility. Third, comment research should distinguish raw frequency, like-weighted analytical salience, and reply-weighted analytical salience.

Practically, brands and platforms should not judge audience response only by whether a video becomes popular or by total interaction volume. For service-performance content such as Haidilao dance videos, circulation management needs to simultaneously attend to immediate entertainment, performer/service appreciation, cringe, employee-labor perception, and judgments about service boundaries. Platform and brand monitoring should also interpret likes and replies separately. High likes do not necessarily represent a general attitude, and high replies do not necessarily represent negative controversy. This recommendation is consistent with the basic distinction in social media engagement research: different interaction indicators correspond to different levels of participation intensity, expression cost, and social meaning (Trunfio and Rossi, 2021; Tenenboim, 2022).

### 4.5 Limitations and future research

The study has six principal limitations. First, the corpora are nonprobability samples; exact collection windows and original selection logs are not documented in the release, and Douyin lacks complete source and original-comment identification. Second, deterministic rules can miss irony, idiom, negation scope, and multilingual referents; category 4 is broader than service quality, and non-text records cannot establish emotional neutrality. Third, the 300-record reference audit overlaps development examples and lacks documented coder-level provenance, so independent validation remains necessary. Fourth, 364 Douyin reply counts are missing; observed-case summaries are primary, and zero replacement is only a sensitivity. Fifth, highly engaged records materially affect weighted categories and stage profiles, and targeted human review was introduced to make slight contextual refinements. Sixth, TikTok models contain only eight source clusters, and all six jackknife intervals include 1. Cumulative interaction counts do not establish reaction timing, individual exposure, or causality. Continued documentation of classifier updates, expanded human validation, and source-complete prospective sampling would strengthen future work.

## 5 Conclusion

This study identifies systematic differences in machine-coded audience appraisal across the sampled Douyin and TikTok comment environments. Other/participatory context is the largest raw category in both corpora (53.5% Douyin, 52.1% TikTok), while performer/service appreciation accounts for 23.2 and 26.9%, and entertainment/humor for 12.9 and 17.6%, respectively. Emotional labor/service boundaries account for 4.6% of Douyin comments and 2.2% of TikTok comments.

Interaction weighting reveals that category frequency and interaction concentration are not equivalent. In Douyin, other/participatory context remains the largest like- and reply-weighted category, while entertainment/humor receives 28.7% of like-weighted and 28.2% of reply-weighted salience. In TikTok, other/participatory context also remains dominant, whereas emotional labor/service boundaries rise from 2.2% of raw comments to 19.9% of like-weighted salience. Likes and replies therefore capture distinct patterns of analytical prominence and are sensitive to highly interacted-with comments.

The pooled sequence analysis describes substantial variation under ordinary interaction weights, but Douyin’s apparent Stage-2 negative peak is largely concentrated in highly replied-to records. TikTok ordered-window coefficients are exploratory: two CR1/*t*(7) intervals exclude 1, whereas all six source-jackknife intervals include 1. No robust cross-platform predictive difference, causal effect, or individual psychological change is established.

The methodological contribution is a reproducible statistical comparison of supplied rule-based labels, raw record composition, interaction-weighted salience, pooled stages, and source-aware TikTok windows. Its usefulness depends on keeping these different units and estimands explicit and on reporting classification and sensitivity limitations alongside point estimates.

These findings describe audience appraisals and recorded interaction patterns in the sampled platform-language environments. They suggest that entertainment, performer appreciation, labor concerns, and service-boundary judgments merit separate examination, while providing insufficient evidence to generalize those patterns to whole platform populations.

## Data Availability

The original contributions presented in the study are included in the article/Supplementary material, further inquiries can be directed to the corresponding author.

## References

[ref1] Cameron A. C. Miller D. L. (2015). A practitioner’s guide to cluster-robust inference. J. Hum. Resour. 50, 317–372. doi: 10.3368/jhr.50.2.317

[ref2] Chen J. Xia S. (2024). Are online users influenced by what other users say? Meta-analyzing the cognitive, emotional, and behavioral impact of online comment valence. Cyberpsychology 18:Article 8. doi: 10.5817/CP2024-5-8

[ref3] Elfenbein H. A. Ambady N. (2002). On the universality and cultural specificity of emotion recognition: a meta-analysis. Psychol. Bull. 128, 203–235. doi: 10.1037/0033-2909.128.2.203, 11931516

[ref4] Fang X. Van Kleef G. A. Sauter D. A. (2019). Revisiting cultural differences in emotion perception between easterners and westerners: Chinese perceivers are accurate, but see additional non-intended emotions in negative facial expressions. J. Exp. Soc. Psychol. 82, 152–159. doi: 10.1016/j.jesp.2019.02.003

[ref5] Ferrara E. Yang Z. (2015). Measuring emotional contagion in social media. PLoS One 10:e0142390. doi: 10.1371/journal.pone.0142390, 26544688 PMC4636231

[ref6] Garcia D. Kappas A. Küster D. Schweitzer F. (2016). The dynamics of emotions in online interaction. R. Soc. Open Sci. 3:160059. doi: 10.1098/rsos.160059, 27853586 PMC5108936

[ref7] Grandey A. A. (2000). Emotion regulation in the workplace: a new way to conceptualize emotional labor. J. Occup. Health Psychol. 5, 95–110. doi: 10.1037/1076-8998.5.1.95, 10658889

[ref8] Grandey A. A. Gabriel A. S. (2015). Emotional labor at a crossroads: where do we go from here? Annu. Rev. Organ. Psychol. Organ. Behav. 2, 323–349. doi: 10.1146/annurev-orgpsych-032414-111400

[ref9] Groth M. Hennig-Thurau T. Walsh G. (2009). Customer reactions to emotional labor: the roles of employee acting strategies and customer detection accuracy. Acad. Manag. J. 52, 958–974. doi: 10.5465/amj.2009.44634116

[ref10] Hilbe J. M. (2011). Negative Binomial Regression. 2nd Edn Cambridge: Cambridge University Press.

[ref11] Hochschild A. R. (1983). The Managed Heart: Commercialization of Human Feeling. Berkeley, CA: University of California Press.

[ref12] Huang A. Zainol Z. (2025). Cross-cultural differences in the effectiveness of short video marketing: a comparative analysis of TikTok and Douyin. Asian People J. 8, 171–187. doi: 10.37231/apj.2025.8.2.827

[ref13] Humphrey N. M. (2023). Emotional labor and employee outcomes: a meta-analysis. Public Adm. 101, 422–446. doi: 10.1111/padm.12818

[ref14] Kaye D. B. V. Chen X. Zeng J. (2021). The co-evolution of two Chinese mobile short video apps: parallel platformization of Douyin and TikTok. Mob. Media Commun. 9, 229–253. doi: 10.1177/2050157920952120

[ref15] Krämer N. C. Neubaum G. Winter S. Schaewitz L. Eimler S. Oliver M. B. (2021). I feel what they say: the effect of social media comments on viewers’ affective reactions toward elevating online videos. Media Psychol. 24, 332–358. doi: 10.1080/15213269.2019.1692669

[ref16] Lazarus R. S. (1991). Emotion and Adaptation. New York, NY: Oxford University Press.

[ref17] Liu M. W. Zhang L. Keh H. T. (2019). Consumer responses to high service attentiveness: a cross-cultural examination. J. Int. Mark. 27, 56–73. doi: 10.1177/1069031X18822968

[ref18] Luo Y. Sornette D. Lera S. C. (2025). Social feedback amplifies emotional language in online video live chats. Commun. Psychol. 3:Article 188. doi: 10.1038/s44271-025-00370-y, 41315718 PMC12753739

[ref19] Mesquita B. Frijda N. H. (1992). Cultural variations in emotions: a review. Psychol. Bull. 112, 179–204. doi: 10.1037/0033-2909.112.2.179, 1454891

[ref20] Papacharissi Z. (2015). Affective Publics: Sentiment, Technology, and Politics. New York, NY: Oxford University Press.

[ref21] Scherer K. R. (2001). “Appraisal considered as a process of multilevel sequential checking,” in Appraisal Processes in Emotion: Theory, Methods, Research, eds. Scherer K. R. Schorr A. Johnstone T. (Oxford: Oxford University Press), 92–120.

[ref22] Stsiampkouskaya K. Joinson A. N. Piwek L. Ahlbom C.-P. (2021). Emotional responses to likes and comments regulate posting frequency and content change behavior on social media: an experimental study and mediation model. Comput. Hum. Behav. 124:Article 106940. doi: 10.1016/j.chb.2021.106940

[ref23] Tenenboim O. (2022). Comments, shares, or likes: what makes news posts engaging in different ways. Soc. Media + Soc. 8:Article 20563051221130282. doi: 10.1177/20563051221130282

[ref24] Trunfio M. Rossi S. (2021). Conceptualising and measuring social media engagement: a systematic literature review. Ital. J. Mark. 2021, 267–292. doi:10.1007/s43039-021-00035-8

[ref25] van Kleef G. A. (2009). How emotions regulate social life: the emotions as social information model. Curr. Dir. Psychol. Sci. 18, 184–188. doi: 10.1111/j.1467-8721.2009.01633.x

