# Supplementary material for: Audience emotional appraisals and interaction-weighted salience of short-video communication in international contexts: evidence from Haidilao dance videos on Douyin and TikTok
Source: Front Psychol. 2026 Oct 16;17:1931735. doi: 10.3389/fpsyg.2026.1931735 (PMC13719608; doi:10.3389/fpsyg.2026.1931735)
Supplement: Supplementary file 1 [file Table_5.DOCX]

# Three_Column_Annotation.py

"""Deterministic multilingual text rules for the formal machine-coded corpus.

No identifier, platform, interaction count or human label is a classifier input.
The completed 300-comment manual validation is documented in
validation_summary.json and does not change these frozen machine outputs.
"""
import argparse,json,re,unicodedata
from pathlib import Path
VERSION='HDL-MACHINE-RULES-20260927-v6.0'
CATEGORIES={1:'Entertainment/Humor',2:'Cringe/Social Discomfort',3:'Curiosity/Spectacle',4:'Performer/Service Appreciation',5:'Emotional Labor/Service Boundaries',6:'Other/Participatory Context'}
RULES=[
('T5_COMPENSATION','labor',r'工资|工資|月薪|薪水|加薪|薪资|薪資|小费|小費|工钱|工錢|底薪|奖金|獎金|\b(?:tips?|tipp(?:ed|ing)|salary|salaries|wages?|paycheck|paid|payed|compensation|underpaid|tippiä)\b|lương|tiền\s*(?:boa|bo)|\bgaji\b'),
('T5_REQUIREMENT','labor',r'被迫|强迫|強迫|逼良为娼|逼良為娼|营业|營業|工伤|工傷|打工人|\b(?:forced|forcing|overworked|underpaid|exploited|exploitation|emotional labor|emotional labour)\b|ép\s+(?:nhảy|buộc)|\b(?:dipaksa|terpaksa)\b'),
('T5_JOB_DEMAND','labor',r'(?:服务员|服務員|员工|員工|服务生|服務生|上班|入职|入職|面试|面試|招聘|兼职|兼職|工作).{0,22}(?:卷|门槛|門檻|全能|多才|辛苦|难|難|累|会跳|會跳|要跳|技能|要求|才艺|才藝)|(?:卷|辛苦|累|难|難).{0,15}(?:服务员|服務員|员工|員工|服務生|服务生)|(?:nhân viên).{0,15}(?:mệt|khổ)|(?:mệt|khổ).{0,15}nhân viên|\b(?:job|staff|worker|waiter|employee)s?\b.{0,25}\b(?:tired|exhausted|require|must|have to)\b|kualifikasi.{0,35}(?:dance|nari)|tuyển.{0,15}(?:dancer|nhân viên)'),
('T5_PERFORMER_SAFETY','safety',r'崴脚|崴腳|扭脚|扭腳|脚扭|腳扭|脚歪|腳歪|脚踝|腳踝|伤到|傷到|trật khớp|lận chân|\bankle\b.{0,20}(?:hurt|broken|better days|break)|\b(?:break|sprain|hurt)\w*\b.{0,15}\b(?:ankle|leg|foot)\b'),
('T2_DISCOMFORT','discomfort',r'尴尬|尷尬|社死|好尬|太尬|挺尬|很尬|尬不尬|尬死|油腻|油膩|油感|恶心|噁心|辣眼|低俗|猥琐|猥瑣|不适|不適|跳的什么玩意|跳得什么玩意|妆好吓人|\b(?:cringe|cringey|cringy|awkward|embarrassing|uncomfortable|disgusting|creepy|repulsive|gross)\b|quê\s+(?:quá|vãi)|ngượng|xấu hổ|\b(?:canggung|memalukan|jijik)\b'),
('T3_INFORMATION','curiosity',r'哪[里裡家个個儿兒]|地址|坐标|座標|这是哪|這是哪|在哪|谁知道|誰知道|什么歌|什麼歌|歌名|什么名字|什麼名字|怎么做到|怎麼做到|怎么火|怎麼火|什么舞|什麼舞|为啥叫|為啥叫|为什么.{0,12}叫|这是啥|這是啥|谁教|誰教|求教程|\b(?:where|which (?:city|outlet|restaurant|branch)|what (?:is|are|song|dance)|who is|song name|song title|tutorial|title of this)\b|ở\s+đâu|tỉnh nào|bài\s+(?:gì|nào)|tên\s+(?:bài|nhạc)|là\s+ai|quản lý.*[?？]|\b(?:di mana|dimana|apa ini|lagu apa|siapa)\b'),
('T3_SURPRISE','spectacle',r'世界奇观|世界奇觀|没想到|沒想到|竟然|原来如此|原來如此|不可思议|不可思議|\b(?:wow|woah|whoa|unbelievable|surprised|surprising|no way|what on earth|what the hell)\b|kaget|ngạc nhiên|😲|😮|😱|\[震惊\]|\[震驚\]'),
('T1_HUMOR','humor',r'哈{2,}|呵{3,}|笑死|笑哭|好笑|搞笑|整活|姨母笑|逗死|鸭叫|鴨叫|\b(?:ha(?:ha)+|he(?:he)+|hhh+|lol+|lmao+|lmfao+|funny|hilarious|amusing|joking|joke|comedy|entertaining|entertainment)\b|\b(?:kkk+|wkwk\w*|haha\w*)\b|buồn cười|hài hước|\blucu\b|ㅋㅋ|哈哈|5555'),
('T1_PLAYFUL','playful',r'恋爱脑|戀愛腦|花痴|妇道|婦道|贪图美色|貪圖美色|迷成|这盛世|這盛世|寡人|秦始皇|竞选宣言|競選宣言|男模.{0,25}(?:性价比|性價比|不起)|夜店.{0,25}(?:对手|對手|海底)|KTV.{0,25}(?:对手|對手|海底)|\b(?:pov|before gta|social credit|ahh|blud|simp)\b|sao.{0,12}đẹp|\bwhy\b.{0,20}\b(?:cute|handsome|beautiful|love)\b'),
('C_SERVICE','service_context',r'服务|服務|服务员|服務員|服务生|服務生|员工|員工|海底捞|海底撈|海底劳|海底勞|客人|顾客|顧客|餐厅|餐廳|餐馆|餐館|\b(?:service|staff|waiter|waitress|worker|employee|customer|restaurant|haidilao|hadilao|haidiliao)s?\b|nhân viên|phục vụ|\b(?:pelayan|karyawan)\b'),
('C_PERFORMANCE','performance',r'跳|舞|科目三|表演|动作|動作|节奏|節奏|丝滑|絲滑|专业|專業|有劲|有勁|\b(?:danc\w*|perform\w*|energetic|footwork|moves|engagement|attitude)\b|nhảy|điệu|\b(?:nari|tarian)\b|เต้น'),
('C_SERVICE_PRAISE','service_praise',r'服务.{0,8}(?:好|棒|赞|讚|绝|絕|热情|熱情)|服務.{0,8}(?:好|棒|讚|絕|熱情)|热情|熱情|敬业|敬業|用心|尽心|盡心|宾至如归|賓至如歸|不让人失望|不讓人失望|\b(?:great|good|excellent|amazing|wonderful|friendly|enthusiastic)\s+(?:customer\s+)?service\b|\b(?:love|appreciate)\b.{0,15}\b(?:service|staff|attitude)\b|\b(?:nice staff|good job|need all worker)\b|phục vụ.{0,12}(?:tốt|nhiệt tình)|pelayanan.{0,12}(?:bagus|baik)'),
('C_VISIT_EXPERIENCE','visit_experience',r'(?:吃饭|吃飯|去吃|去这吃|去這吃|消费|消費|餐桌|现场|現場).{0,35}(?:跳|表演|助兴|助興|好看)|(?:跳|表演|助兴|助興).{0,35}(?:吃饭|吃飯|去吃|消费|消費)|ăn cơm.{0,12}(?:ngon|vui)|\b(?:experience|dancing service)\b.{0,30}\b(?:restaurant|good|love|enjoy)\b'),
('P_POSITIVE','positive',r'喜欢|喜歡|好看|帅|帥|好棒|真棒|不错|不錯|厉害|厲害|好酷|最酷|丝滑|絲滑|最能|最好|很自然|有节奏感|有節奏感|跳得好|跳的好|爱了|愛了|爱看|愛看|\b(?:love|like|enjoy|good|great|nice|beautiful|handsome|cute|amazing|awesome|wonderful|excellent|cool|masterpiece|tuff)\b|đẹp|thích|\bmê\b|\bhay\b|đỉnh|cuốn|nhảy giỏi|\b(?:ganteng|bagus|suka|keren|cantik)\b|สวย|หล่อ|เท่ห์|잘생|멋있|かわいい|かっこいい'),
('P_NEGATIVE','negative',r'讨厌|討厭|难看|難看|不喜欢|不喜歡|不好看|无聊|無聊|吓人|嚇人|油腻|油膩|恶心|噁心|尴尬|尷尬|\b(?:hate|awful|terrible|horrible|bad|boring|disgusting|cringe|awkward|uncomfortable|ugly)\b|\bchán\b|\bxấu\b|tệ quá|\b(?:jelek|bosan|buruk)\b'),
('P_EXTREME_POS','extreme_positive',r'帅死|帥死|帅炸|帥炸|帅到爆|帥到爆|帅翻|帥翻|帅呆|帥呆|绝了|絕了|爱死|愛死|太.{0,4}(?:帅|帥|好看|喜欢|喜歡)|最.{0,8}(?:帅|帥|好看|好|酷)|救命.{0,25}(?:看|帅|帥|喜欢|喜歡)|\b(?:obsessed|addicted|masterpiece|absolutely love|so damn good|love this so much)\b|đẹp.{0,12}(?:xỉu|quá trời|nhất)|đỉnh|tuyệt vời'),
('P_EXTREME_NEG','extreme_negative',r'恶心死|噁心死|尬死|尴尬死|尷尬死|尴尬的一|尷尬的一|恨死|受不了|看不下去|\b(?:absolutely hate|utterly disgusting|unbearable|horrifying|repulsive)\b'),
('I_AMPLIFIER','amplifier',r'太|真(?:的)?|非常|特别|特別|超级|超級|简直|簡直|巨|极其|極其|\b(?:very|really|so|super|extremely|absolutely|incredibly|damn|literally)\b|\bquá\b|rất|cực|nhất|\bbanget\b|มาก'),
('I_MITIGATOR','mitigator',r'有点|有點|稍微|还行|還行|一般|\b(?:slightly|a bit|kind of|sort of|okay|ok)\b|hơi|\bagak\b'),
]
CONFIG=[dict(id=i,role=role,pattern=p) for i,role,p in RULES]
\# Text-only development review identified idiomatic job demands, deliberate
\# parody, rhetorical appraisal and multilingual enjoyment missed by the draft.
CONFIG.extend([
 dict(id='A_APPEARANCE',role='appearance',pattern=r'帅|帥|好看|颜值|顏值|颜质|顏質|漂亮|可爱|可愛|英俊|\b(?:handsome|cute|beautiful|pretty|gorgeous|attractive|hot|good[- ]looking)\b|đẹp trai|đẹp|đz|dễ thương|\b(?:ganteng|cantik)\b|หล่อ|สวย|잘생|かわいい|かっこいい'),
 dict(id='D_JOB_CONTEXT',role='labor',pattern=r'没点才艺.{0,18}面试|沒點才藝.{0,18}面試|鞋坏了.{0,12}包换|鞋壞了.{0,12}包換|后厨.{0,12}洗|後廚.{0,12}洗|上班屈才|教.{0,10}员工.{0,12}跳|\bteaching our staff\b|\bwork\b.{0,45}\b(?:bisa nari|tahan malu)\b|一个月多少个|一個月多少個'),
 dict(id='D_SERVICE_APPRAISAL',role='service_praise',pattern=r'(?:服务生|服務生|员工|員工|服务员|服務員).{0,12}(?:素质|素質|敬业|敬業)|(?:不让人失望|不讓人失望)|(?:最好的海底.{0,3}版本)|有这服务.{0,15}去吃'),
 dict(id='D_ENJOYMENT',role='enjoyment',pattern=r'爱了|愛了|爱看|愛看|好喜欢|好喜歡|看了.{0,8}(?:遍|分钟|分鐘).{0,10}(?:还想看|還想看)|(?:看了|看过|看過).{0,6}(?:十遍|七八遍)|拒绝不了|拒絕不了|跳得.{0,5}好|跳的.{0,5}好|\b(?:love this|enjoy this|entertaining|obsessed|addicted|masterpiece)\b|j[’\']aime|nghiện|nhảy.{0,12}(?:giỏi|đẹp|ưng)|điệu nhảy.{0,15}(?:mê|đỉnh)|\bmê\b.{0,12}điệu|ดูไม่จบ|เต้น.{0,12}(?:สวย|เท่)|\bfootwork\b.{0,20}\bgood\b'),
 dict(id='D_POSITIVE_IDIOM',role='positive',pattern=r'松弛感|松弛|无人超越|無人超越|这个牛|這個牛|不让人失望|不讓人失望|好吃|有劲|有勁|有点东西|有點東西|颜值.{0,6}高|顏值.{0,6}高|颜质.{0,6}高|顏質.{0,6}高|跳得.{0,5}好|跳的.{0,5}好|j[’\']aime|đz|\bưng\b|nghiện'),
 dict(id='D_INQUIRY',role='curiosity',pattern=r'去哪了|那的海底|一个问题|一個問題|有一個問題|到底.{0,8}什么|到底.{0,8}什麼|怎么没|怎麼沒|是.*[?？]$|có\s+(?:ko|không)\s+ạ|bên nào'),
 dict(id='D_PARODY',role='playful',pattern=r'夜店.{0,30}(?:败给|敗給|对手|對手)|KTV.{0,30}(?:败给|敗給|对手|對手)|前男友.{0,50}海底|服务员.{0,15}捞月亮|服務員.{0,15}撈月亮|科目三考试.{0,20}教程|科目三考試.{0,20}教程|\bsocial credit\w*\b'),
])
def clean(text):
 s=unicodedata.normalize('NFKC',str(text or '')).strip()
 if s.startswith(("'+","'=","'-","'@")):s=s[1:]
 s=re.sub(r'\[(?:用户(?:标识)?|账号|用戶(?:標識)?|帳號)已隐去\]|(?<!\w)@[\w.\-]+',' ',s)
 return re.sub(r'\s+',' ',s).strip()
def classify(text,rules=None):
 s=clean(text); flags=[];evidence=[];matched=[]
 media=re.sub(r'\[(?:Photo|Sticker|Image|图片|貼圖|贴图)\]','',s,flags=re.I).strip()
 no_text=not str(text or '').strip() or (not media and bool(re.search(r'\[(?:Photo|Sticker|Image|图片|貼圖|贴图)\]',s,re.I)))
 if no_text:return dict(valence=3,intensity=1,category=6,category_name=CATEGORIES[6],strict_service_category=6,appreciation_subtype='Not applicable',appearance_referent='Not evaluable',rules='STRUCT_NO_TEXT',evidence='No accessible text; structural placeholder',flags='Not an inferred affective response',confidence='Not evaluable',version=VERSION,text_evaluable=0)
 \# Emoji aliases are parsed separately from verbal cues. In particular,
 \# [尬笑] is not equivalent to an explicit verbal statement of discomfort.
 verbal=re.sub(r'\[[^\]]+\]',' ',s)
 hits={}
 for r in (rules or CONFIG):
 target=s if r['role']=='spectacle' else verbal
 ms=list(re.finditer(r['pattern'],target,re.I))
 valid=[]
 for m in ms:
 pre=target[max(0,m.start()-22):m.start()]
 negated=bool(re.search(r"(?:\b(?:not|never|no|don't|dont|don’t|isn't|isn’t)\s+(?:really\s+|that\s+|too\s+)?|(?:不|没|沒)(?:太|很|是)?|(?:không|khong|ko|chẳng|chả)\s+|(?:tidak|nggak|gak)\s+)$",pre,re.I))
 if negated and r['role'] in ['positive','negative','discomfort','extreme_positive','extreme_negative']:
 flags.append('Local negation reverses evaluative direction')
 if r['role']=='positive':hits.setdefault('negated_positive',[]).append(m.group())
 elif r['role'] in ['negative','discomfort']:hits.setdefault('negated_negative',[]).append(m.group())
 continue
 if r['role']=='positive' and m.group().lower()=='like' and re.search(r'\b(?:looks?|feels?|sounds?|just)\s*$',pre,re.I):continue
 valid.append(m.group())
 if valid:
 hits.setdefault(r['role'],[]).extend(valid);matched.append(r['id']);evidence.append(r['id']+': '+', '.join(dict.fromkeys(valid))[:220])
 def has(role):return bool(hits.get(role))
 laughter=re.findall(r'[😂🤣😆😄😁😃😝🤪]|\[(?:笑哭|呲牙|大笑|偷笑|憨笑|奸笑)\]',s)
 affection=re.findall(r'[🥰😍😘❤♥💖💕💗💓💝💞💜💙💚💛🧡🤤👍👏🫰🌹]|\[(?:爱心|愛心|比心|送心|色|舔屏|赞|贊|鼓掌|玫瑰|亲亲|親親|666)\]',s)
 distress=re.findall(r'[😡🤬🤮👎😢😞]|\[(?:发怒|發怒|愤怒|憤怒|流泪|流淚|吐)\]',s)
 ambiguous=re.findall(r'[😭💀😳😅🥺🙃💔🥀]|\[(?:泪奔|淚奔|泣不成声|泣不成聲|捂脸|捂臉|尬笑)\]',s)
 if ambiguous:flags.append('Ambiguous emoji interpreted with verbal context; no fixed negative polarity')
 if laughter:matched.append('EMOJI_LAUGHTER');evidence.append('EMOJI_LAUGHTER: '+''.join(laughter[:8]))
 if affection:matched.append('EMOJI_APPRECIATION');evidence.append('EMOJI_APPRECIATION: '+''.join(affection[:8]))
 if distress:matched.append('EMOJI_DISTRESS');evidence.append('EMOJI_DISTRESS: '+''.join(distress[:8]))
 self_safety=bool(re.search(r'(?:我|俺).{0,10}(?:学|學|跳|试|試|扭|练|練)|\b(?:my|i would|i tried)\b|tui sợ|tôi sợ',verbal,re.I))
 labor=has('labor') or (has('safety') and not self_safety)
 discomfort=has('discomfort') or has('negated_positive')
 positive=has('positive') or has('extreme_positive') or has('negated_negative') or has('enjoyment')
 negative=has('negative') or has('extreme_negative') or has('negated_positive') or has('discomfort')
 \# A concession explicitly ending in praise gives the final appraisal
 \# direction, but remains flagged for review rather than silently resolved.
 if re.search(r'(?:但|不过|不過|可是).{0,25}(?:帅|帥|好看|喜欢|喜歡)',verbal):
 positive=True;negative=False;discomfort=False;flags.append('Contrastive final-clause praise')
 service=(has('service_praise') and has('service_context') and not negative) or (has('service_context') and has('performance') and positive and not negative) or (has('visit_experience') and positive and not negative)
 curiosity=has('curiosity') or has('spectacle')
 rhetorical=positive and bool(re.search(r'为啥.{0,10}帅|為啥.{0,10}帥|怎么.{0,10}帅|怎麼.{0,10}帥|sao.{0,12}đẹp|why.{0,20}(?:cute|handsome|love|beautiful)',verbal,re.I))
 if rhetorical and not has('curiosity'):curiosity=False
 if has('playful') and not has('curiosity'):curiosity=False
 entertainment=has('humor') or has('playful') or has('enjoyment') or bool(laughter) or (has('performance') and positive)
 themes=[(5,labor),(2,discomfort),(4,service),(3,curiosity),(1,entertainment)]
 category=next((c for c,t in themes if t),6)
 strict_service_category=category
 \# v4.1 is a disclosed construct expansion requested after v4 evaluation.
 \# Appearance admiration is NEVER called evidence of service quality.
 \# Explicit performer reference or an elliptical appraisal of the video
 \# may enter 4b; self/third-party/object descriptions are conservatively
 \# excluded. This rule is not selected by an audit agreement target.
 other_referent=bool(re.search(r'我(?:也|才|最|很|真|太|好|不)?(?:帅|帥|好看|漂亮|可爱|可愛)|我(?:的)?(?:老公|老婆|孩子|女儿|女兒|儿子|兒子|朋友)|\b(?:i am|i\x27m|my (?:boyfriend|girlfriend|wife|husband|child|friend))\b|(?:衣服|裙子|鞋子|食物|菜|饭|飯|装修|裝修|风景|風景).{0,8}(?:好看|漂亮|可爱|可愛)|\b(?:food|song|shoes|dress|outfit|scenery|restaurant)\s+(?:is |looks? )?(?:so |very )?(?:beautiful|cute|pretty|hot)\b',verbal,re.I))
 explicit_performer=bool(re.search(r'小哥|帅哥|帥哥|小姐姐|小哥哥|男生|女生|这个人|這個人|他|她|\b(?:he|she|him|her|guy|boy|girl|dancer|waiter|staff)\b|anh|cậu|bạn|abang',verbal,re.I))
 elliptical=has('appearance') and len(verbal.strip())<=100 and not has('curiosity')
 appearance=has('appearance') and positive and not negative and not other_referent and (explicit_performer or elliptical)
 appearance_referent='Explicit performer reference' if appearance and explicit_performer else 'Elliptical video-context reference; inferred' if appearance else 'Not established'
 appreciation_subtype='4a Service/staff-performance appraisal' if category==4 else 'Not applicable'
 if appearance and category not in [2,3,5]:
 if category==4:appreciation_subtype='4a+4b Service/performance and appearance appraisal'
 else:category=4;appreciation_subtype='4b Performer appearance admiration'
 matched.append('A_PERFORMER_APPRECIATION');evidence.append('A_PERFORMER_APPRECIATION: '+appearance_referent)
 flags.append('Appearance appreciation is not an observed measure of service quality')
 \# General positive reactions to a performance belong to a broad
 \# appreciation frame. This includes emoji-only appreciation but does
 \# not assert a particular appearance trait or service quality.
 general_appreciation=(positive or bool(affection)) and not negative and not distress
 if category==6 and general_appreciation:
 category=4;appreciation_subtype='4c General performance appreciation'
 matched.append('A_GENERAL_APPRECIATION');evidence.append('Positive verbal or emoji appraisal in the service-dance comment context')
 flags.append('General appreciation; specific referent and service-quality appraisal are not established')
 \# Pure playful/ambiguous reaction emoji can indicate amused participation
 \# only when laughter or a verbal playful frame is also present.
 if sum(bool(t) for _,t in themes)>1:flags.append('Multiple appraisal frames; explicit labor/discomfort/service/inquiry precede amusement')
 \# Verbal appraisal dominates ambiguous emoji. Explicit opposite verbal
 \# appraisals are mixed. Theme membership never determines valence.
 if positive and negative:valence=3;flags.append('Mixed verbal appraisal')
 elif positive:valence=5 if has('extreme_positive') or (has('amplifier') and has('positive')) else 4
 elif negative:valence=1 if has('extreme_negative') else 2
 elif distress and not (affection or laughter):valence=2
 elif (affection or laughter or has('humor')) and not distress:valence=4
 else:valence=3
 \# Intensity anchors integrate semantic strength and expressive channels.
 \# A clear evaluation is moderate even without repetition. Repetition
 \# amplifies affective evidence but is insufficient on neutral text alone.
 punct=bool(re.search(r'[!！?？]{2,}',s));caps=bool(re.search(r'\b[A-Z]{6,}\b',verbal))
 repetition=bool(re.search(r'([^\W\d_])\1{2,}',verbal,re.UNICODE))
 extreme=has('extreme_positive') or has('extreme_negative') or bool(re.search(r'笑死|救命|受不了|\b(?:screaming|dying|obsessed)\b|xỉu',verbal,re.I))
 affect=positive or negative or has('humor') or has('playful') or bool(laughter or affection or distress or ambiguous)
 expressive=len(laughter)+len(affection)+len(distress)+len(ambiguous)
 if not affect:intensity=2 if has('spectacle') or punct or caps else 1
 elif has('mitigator') and not extreme and expressive<=1:intensity=2
 else:
 intensity=3
 if has('amplifier') or extreme or caps or repetition or punct or expressive>=3:intensity=4
 if extreme and (has('amplifier') or repetition or punct or expressive>=2):intensity=5
 elif expressive>=6 and (affection or laughter or distress):intensity=5
 elif (has('amplifier') or caps) and (punct or repetition) and expressive>=2:intensity=5
 if re.search(r'\b(?:sarcasm|sarcastic|ironic)\b|反讽|反諷|呵呵',s,re.I):flags.append('Possible irony; semantic review recommended')
 if category==6:flags.append('Residual context or unresolved referent; no supported primary theme')
 confidence='High contextual specificity' if category in [2,4,5] and not any('Mixed' in f or 'Contrastive' in f for f in flags) else 'Moderate contextual specificity' if category in [1,3] else 'Low or unresolved theme specificity'
 if appearance and not explicit_performer:confidence='Moderate specificity; referent inferred from video context'
 return dict(valence=valence,intensity=intensity,category=category,category_name=CATEGORIES[category],strict_service_category=strict_service_category,appreciation_subtype=appreciation_subtype,appearance_referent=appearance_referent,rules='; '.join(matched) or 'CONTEXT_FALLBACK',evidence='; '.join(evidence)[:3000],flags='; '.join(dict.fromkeys(flags)) or 'Automatic contextual estimate',confidence=confidence,version=VERSION,text_evaluable=1)
if __name__=='__main__':
 p=argparse.ArgumentParser();p.add_argument('--text',required=True);p.add_argument('--rules');a=p.parse_args();print(json.dumps(classify(a.text,json.loads(Path(a.rules).read_text()) if a.rules else None),ensure_ascii=False,indent=2))
